# Hot Topics in Cellular Neuropathology

**DOI:** 10.3389/fncel.2020.627494

**Published:** 2020-12-02

**Authors:** Dirk M. Hermann

**Affiliations:** Department of Neurology, University Hospital Essen, University of Duisburg-Essen, Essen, Germany

**Keywords:** neurology, translational neuroscience, neuropathology, applied neuroscience, clinical neuroscience, therapy

Methodological developments have greatly instructed progress in biomedicine. The discovery of the CRISPR/Cas9 technology by Charpentier and Doudna in 2012, which was awarded with the Nobel Prize in Chemistry in 2020, is a strong example here, because, by enabling genomic editing, it has become possible to introduce gene sequences into genomes with extremely high precision in extremely fast and simple ways (Jinek et al., 2012). The CRISPR/Cas9 technology was a side product of research on Streptococcus pyogenes, in which Charpentier discovered a previously unknown molecule, tracrRNA, which is part of the bacterium's defense mechanism that disarms viruses by cleaving their DNA (Deltcheva et al., 2011). After modifying the bacteria's scissors in a test tube and simplifying their molecular components so they were easier to use, Charpentier and Doudna reprogrammed the scissors so that they can cut any DNA molecule at a predetermined site (Jinek et al., 2012). The application to eukaryotic cells enables hitherto unprecedented possibilities in gene editing, which fundamentally enriches cellular physiology and pathology. Hence, CRISPR/Cas9 has become a cornerstone in biotechnology in the last 8 years. Today, the CRISPR/Cas9 technology is used all over the world in biosciences, and it opens fascinating perspectives in Cellular Neuropathology too.

Scientific breakthroughs are strongly facilitated by technological advances. They are accompanied by changes in human mindsets, which enable the proper interpretation of the observations made. The discovery of heliocentrism by Copernicus (1543) followed the mind opening at the transition from medieval to modern ages. Only a few decades later the development of telescopes provided proofs about the orbits of planets and their moons, which rejected that the earth was in the center of this world. The evolution of species that was discovered by Darwin (1859) was a consequence of intellectual upraise in the age of enlightenment. It was inseminated by developments of microscopy in the 17th and 18th century and was a consequence of the ability of man to appropriately categorize and interpret research findings. The decoding of deoxyribonucleic acid by Watson and Crick (1953) would have been inconceivable without preceding developments in X-ray crystallography by Rosalind Franklin. It was build up on concepts in organic chemistry evolving at that time and was a starting point of modern genomics. Collectively, the preceding discoveries enabled paradigm changes overcoming limits of imagination by applying innovative methods, raising the right questions, designing appropriate experiments and interpreting research findings in innovative ways.

Compared with the research areas above, neurosciences are still a young research field, founded by developments in histochemistry and electrophysiology in the closing 19th century that established a basis for brain research activities. As in other research fields, subsequent progress in neurosciences was not linear, and methodological advances still occur stepwise. Developments in molecular biology and genetics accelerated developments in neurosciences at the offspring of the new millennium, enabling understandings of the molecular mechanisms controlling cellular functions in the healthy and the injured brain. Unfortunately, in many disease areas these insights still did not translate into the development of disease therapies. Very recently, advances in neuroimaging (two-photon microscopy, superresolution microscopy, lightsheet microscopy) inspired neurosciences by the possibility of imaging neurobiological and neuropathological processes at the nanoscopic level *in vivo* in real-time. At present, bioinformatics (network analysis, deep learning algorithms, computational models) inseminate neuroimaging, electrophysiology, molecular biology and pharmacology, empowered by the possibility of handling big data, utilizing information available and predicting structure-function relationships at the subcellular, cellular and tissue level, which allows the reevaluation of research data in new contexts and may lead to the identification of disease biomarkers or treatment targets, via which biological processes can be modified. To which degree these new techniques will change our research landscape and may ultimately result in the implementation of new therapies, remains to be shown.

Innovation in Cellular Neuropathology is specifically challenging, since discoveries take place at the interface between cellular and clinical neurosciences. Processes and mechanisms typically range from the molecular to the subcellular, cellular and microcircuit levels. Disease processes are amenable using structural and functional readouts, the latter having greater impact on disease severity than the former ones. Progress in Cellular Neuropathology strongly depends on the in depth understanding of cellular and molecular biology, appropriate disease models and innovative research techniques, which need to be employed in ways allowing us to answer clinically relevant questions. The number of ways to tackle biomedical problems is huge, and the number of unmet needs is high. Inspiring research requires a genuine sense of the right methodology applied to a given biomedical problem at a time. Successful research requires that researchers follow hitherto untrodden paths, maintain their alertness for unexpected observations and possess the ability of putting research findings in new contexts. This intellectual capacity alone carries the potential of changing research landscapes.

In an effort to identify the most promising ideas and concepts, the Cellular Neuropathology section of Frontiers in Cellular Neurosciences launches a new platform, the Hot Topics Hub. Within this platform, this journal aims to search for hot topics in the Cellular Neuropathology field, which carry landscape-changing potential, push our understanding of neurological diseases, broaden imagination horizons and may expand current diagnostic or therapeutic possibilities. Papers published in the Hot Topics Hub may include papers introducing new technologies, new disease models or new ways of data analysis that can be used for studying open questions in Cellular Neuropathology. Papers published in this platform may discuss systematic errors in research conception and analysis or present critical reflections on methodological or interpretational constraints that impede progress in the field and prevent proper understandings. Ideas to overcome these bottlenecks will highly be welcome. In this search for the best concepts and thoughts, Original research, Perspectives and Opinions will be accepted. Papers will be judged based on stringency, originality and innovation potential. Authors of excellent papers may be invited to host Research Topics. Outstanding papers will be featured in an editorial.

## Author Contributions

The author confirms being the sole contributor of this work and has approved it for publication.

## Conflict of Interest

The author declares that the research was conducted in the absence of any commercial or financial relationships that could be construed as a potential conflict of interest.
